# Wear Status Monitoring Method of Milling Cutter Under Variable Working Conditions Based on Transfer Learning and Lightweight SqueezeNet Model

**DOI:** 10.3390/s26123835

**Published:** 2026-06-16

**Authors:** Zhaohui Deng, Zhiwu Liu, Da Liu, Rongjin Zhuo, Xiao Yang, Rong Liu

**Affiliations:** Institute of Manufacturing Engineering, Huaqiao University, Xiamen 361021, China; 24011080012@stu.hqu.edu.cn (Z.L.); 22014080053@stu.hqu.edu.cn (D.L.); zhuorj@126.com (R.Z.); whyx97@stu.hqu.edu.cn (X.Y.); 25014088025@stu.hqu.edu.cn (R.L.)

**Keywords:** milling cutter, tool wear status monitoring, variable working conditions, transfer learning, lightweight SqueezeNet model

## Abstract

In the existing tool wear status monitoring process, the difference in the distribution of tool wear signal characteristics under different processing conditions leads to insufficient generalization of the model and poor accuracy of wear status recognition. Aiming at the problem, a method for monitoring the wear status of milling cutters under variable working conditions based on transfer learning and a lightweight SqueezeNet model is proposed. Firstly, the continuous wavelet transform (CWT) is employed to realize the conversion of the raw vibration signal to a time–frequency energy diagram to completely preserve the joint feature distribution of the vibration signal in the time and frequency dimensions. Secondly, based on the phased transfer learning strategy and the lightweight SqueezeNet, a monitoring model of the wear status of the milling cutter under variable working conditions is established, which realizes the adaptive and accurate identification of the wear status of the milling cutter under different milling conditions. Finally, comparative experiments were performed using three groups of vibration signals under different milling condition as the model inputs. As demonstrated by the experimental results, the recognition accuracy of the test set of the proposed monitoring model under variable working conditions can reach 94.583%, which is higher than the 91.133% of the LSTM-DBO-SVM model, which proves the accuracy and feasibility of the presented approach under variable working conditions.

## 1. Introduction

As the core processing part of the machining link under the intelligent manufacturing system, the operational condition of the tool has a direct bearing on the quality and efficiency of manufacturing processes [1,2,3]. Tool wear and breakage are the main causes of tool failure, which will not only degrade the surface quality and dimensional accuracy of the workpiece but also lead to resource waste and even result in permanent damage to the machine tool [4]. According to statistics, 20% of the machine downtime during the machining process is caused by the wear and failure of the tool [5], and 3–12% of production costs are associated with the wear status of the tool and its replacement schedule. Deploying a tool wear status monitoring system can reduce machine downtime by nearly three-quarters, cut maintenance costs by 30%, and significantly improve production efficiency by 10–50% [6]. This highlights that real-time monitoring of the wear status of the tool during machining operations and prompt tool replacement are of critical importance for lowering production costs, enhancing operational efficiency, and elevating product quality.

Currently, extensive research on tool wear monitoring has been conducted by numerous scholars, and these monitoring approaches can be categorized into direct and indirect methods [7,8,9,10]. Among them, the direct method is accomplished mainly through direct measurement, radiation monitoring [11], resistance monitoring [12] and various image processing-based techniques [13] to directly measure the wear change in the measured tool after processing for a period of time. The advantage of this method is that the operation is simple and direct, the technical development has been basically improved, and higher monitoring accuracy can be obtained. The disadvantage is that its specific operation is easily limited by the processing conditions, and it is challenging to realize online monitoring of tool wear status. The indirect method is built upon signal acquisition, processing technologies and an artificial intelligence algorithm. By collecting signal data associated with tool wear, such as cutting force, vibration, power, acoustic emission, and spindle current in the machining process [14,15], the mapping correlation between signal features and tool wear status is established to monitor the tool wear status. It is also the most frequently studied and applied method.

With the rapid advancement of artificial intelligence technology, tool wear monitoring methods based on deep learning have attracted extensive attention from scholars. For example, Li et al. [16] presented an integrated convolutional neural network model combined with independent component analysis based on audio signals, which realized intelligent and accurate identification of the wear level of milling cutters. Xue et al. [17] improved the performance of the tool wear recognition model by introducing an attention mechanism implemented by one-dimensional convolution operations in the convolutional neural network. The excellence of the model is verified by using the public dataset. It is found that the prediction accuracy is improved by 4% and 5%, respectively, compared with the state-of-the-art results reported in existing studies on the T1 and T3 datasets. Abdeltawab et al. [18] proposed a hybrid deep learning framework for milling tool wear status recognition by combining maximum overlap discrete wavelet transform, a convolutional neural network (CNN) and a bidirectional long short-term memory algorithm, which divided the tool wear status into five categories. Comparative experiments demonstrate that the proposed model delivers superior recognition performance, and its overall test accuracy is 94.07%. It can be seen that the deep learning algorithm has been extensively applied in tool wear status monitoring. However, due to the distribution difference in tool wear signal characteristics under different working conditions, the assumption of independent and identical distribution of machine learning input data is broken, so that the reliable identification of tool wear status by most of the existing models is limited to a single machining condition. However, because the distribution difference in tool wear signal characteristics under different machining conditions does not conform to the assumption that the input data of machine learning are independent and identically distributed, most of the existing models are limited to a single machining condition for the accurate identification of tool wear status.

Therefore, some scholars have constructed a tool wear status monitoring model based on the transfer learning method, such as Yan et al. [19], who presented a feature fusion architecture combining deep learning and traditional signal processing. Through the collaborative optimization of a convolutional neural network and classical signal processing methods, the adaptive representation learning of multi-source sensor signals is realized. Based on the transfer learning framework, the backbone network parameters are frozen, and two fully connected layers are incorporated to effectively solve the model adaptability problem stemming from variations in cutting parameters. Wang et al. [20] proposed a cross-working-condition tool condition monitoring method based on transfer learning. Domain adaptation of the transfer learning model was achieved by integrating the Deep CORAL algorithm into the last feature extraction layer of the deep extreme learning machine. On this basis, minimized center loss was introduced to improve the intra-class compactness of the model. It can be seen that the existing tool wear status monitoring model based on the transfer learning method has some limitations: Firstly, the influence of the incomplete characterization of the tool wear signal distribution on model generalization in input data. Secondly, over-fitting and training timeout problems were caused by the over-complicated structure of the pre-training model and too many network parameters.

Therefore, to address the aforementioned problems, this work proposes a method for monitoring the wear status of milling cutters under variable working conditions by combining the staged transfer learning strategy and the lightweight SqueezeNet model. Firstly, CWT is adopted to convert the collected one-dimensional time-series vibration data into a 2D time–frequency diagram to fully retain the joint feature distribution of the signal in both the time and frequency domains. Secondly, based on a transfer learning strategy and a lightweight SqueezeNet, the wear status monitoring model of the milling cutter under variable working conditions is constructed. Finally, the whole life wear experiment of the milling cutter is carried out. Taking the vibration signals under three different milling conditions as input, the performance of the presented model and the LSTM-DBO-SVM method under variable working conditions is compared and analyzed.

## 2. Relate Work

### 2.1. Continuous Wavelet Transform

A time–frequency diagram is an important tool to characterize the joint distribution characteristics of energy density of one-dimensional time-series signals mapped onto a two-dimensional time–frequency domain plane. Compared with the time-series signal, it has a more comprehensive description of the characteristics of the original signal and can provide a feature expression space with clearer physical meaning for input to deep learning models [21]. It is often used to process and analyze non-stationary and non-linear signal data under complex working conditions, such as machining chatter and tool wear. The CWT is one of the main methods to obtain the time–frequency diagram. It realizes the dynamic focusing of different signal frequency bands through the continuous change in scale factor and translation factor, so as to accurately describe the local detail time–frequency features of the signal, that is, in high-frequency signal bands, a narrow time window is employed to enhance time resolution, while in low-frequency signal bands, a wider time window is utilized to improve frequency resolution [22]. Compared with the Discrete Wavelet Transform (DWT), the continuous scale factor of CWT can avoid the problem of binary discretization data distortion and improve the scale division accuracy under high-frequency signal bandwidth.

In the selection of the wavelet basis function, this work uses the complex Morlet wavelet as the basis function for CWT, which is essentially a complex form of the modulated Gaussian function, which is mathematically expressed as:

(1) $$\psi (t)=\frac{1}{\sqrt{\pi F_{b}}}e^{2j\pi F_{c}t}e^{-\frac{t^{2}}{F_{b}}}$$

Among them $F_{b}$ is the bandwidth parameter, and $F_{c}$ is the center frequency. Specifically, the bandwidth parameter $F_{b}$ mainly affects the resolution of the wavelet across both the time and frequency domains. As $F_{b}$ increases, the wavelet’s frequency resolution improves while its time resolution decreases. On the contrary, when it decreases, the frequency resolution of the wavelet decreases and the time resolution increases. The center frequency $F_{c}$ mainly affects the modulation frequency of the wavelet. When its value is small, the wavelet is suitable for analyzing low-frequency signals. When the value is large, it is more suitable for the analysis of high-frequency components.

For a given signal x(t), the CWT expression can be obtained by scaling x(t) with a complex Morlet wavelet as follows [23]:

(2) $$W_{\psi }(a,b)=\int_{\infty }^{-\infty }x\left(t\right)\cdot \psi_{a,b}\left(t\right)dt$$

Among them, a is the scale of the wavelet; b is time translation; both are real numbers. In a finite space, the calculation process of generating sub-wavelet $\psi_{a,b}\left(t\right)$ by wavelet basis ψ(t) is as follows:

(3) $$\psi_{a,b}\left(t\right)=\frac{1}{\sqrt{a}}\psi \left(\frac{t-b}{a}\right)$$

The red-green-blue (RGB) representation of the time–frequency diagram enables more comprehensive mining of frequency-domain characteristics within the signal, while also offering strong anti-interference capabilities. The time–frequency conversion process does not require any external intervention, and the input and output can be completed only according to the conversion formula, which can avoid human interference, to a certain extent, realize the automatic recognition of the time–frequency map and the deep feature learning of SqueezeNet, and then improve the recognition accuracy of the wear status of the milling cutter. Therefore, this work uses the RGB continuous wavelet transform-derived time–frequency diagram as the input to the SqueezeNet model.

### 2.2. SqueezeNet Model

The SqueezeNet model is a lightweight convolutional neural network model proposed by Professor Han’s research team at Berkeley and Stanford University in 2016 [24]. The core design concept is to maintain the accuracy of the model and operational efficiency while drastically reducing the number of network parameters. Compared with the conventional AlexNet convolutional neural network, the structure of SqueezeNet is more efficient and compact. It can reduce the model parameters to 1/50 of AlexNet while maintaining the model performance similar to AlexNet and greatly improving the model parameter scale and space occupation.

The SqueezeNet model has the following three characteristics [25]:

- By replacing some 3 × 3 convolution kernels of the convolutional neural network with 1 × 1 convolution kernels, the total volume of network parameters is reduced, and feature channels are compressed via 1 × 1 convolution to control the expansion rate of the intermediate feature map’s channel dimensions.
- Global average pooling is adopted to replace the traditional fully connected layer, and the position of the pooling layer in the network is delayed to retain the larger size of the feature map to improve the feature expression ability.
- Convolutional layers are substituted with Fire modules, and the network’s expression ability is enhanced by multi-scale feature fusion, which allows the network to shrink the model parameter scale while maintaining strong feature representation abilities.

**Figure 1:**
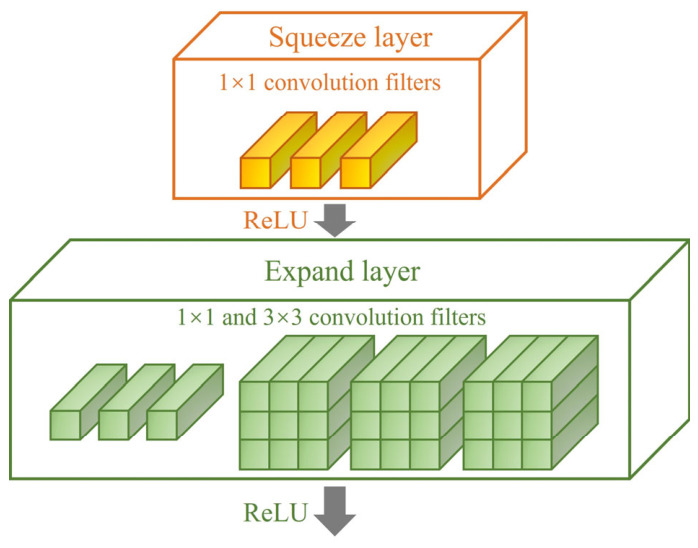
The structure diagram of Fire module.

The specific architecture of the Fire module is illustrated in Figure 1, which is mainly composed of two parts: the Squeeze Layer and the Expand Layer. The count of convolution kernels in the compression layer is smaller than that in the expansion layer, which restricts the volume of input channels entering the 3 × 3 convolution kernels, thereby reducing the number of model parameters.

Then, taking the dimensions of the input feature map as given, the computational flow of the Fire module can be formulated as:

Step 1: The compression layer employs 1 × 1 convolution kernels for channel dimensionality reduction:

(4) $$F_{squeeze}=f\left(W_{s1}\times X+b_{s1}\right)$$

In the formula, $W_{s1}\in R^{1\times 1\times C_{in}\times s_{1\times 1}}$ is the convolution kernel weight, f(·) is the ReLU activation function, and $s_{1\times 1}$ is the number of output channels of the compression layer.

Step 2: The expansion layer performs 1 × 1 and 3 × 3 convolutions in parallel.

(5) $$F_{expand1}=f\left(W_{e1}\times F_{squeeze}+b_{e1}\right)$$

(6) $$F_{expand3}=f\left(W_{e3}\times F_{squeeze}+b_{e3}\right)$$

In the formula, $W_{s1}\in R^{1\times 1\times s_{1\times 1}\times e_{1\times 1}}$ is the convolution kernel weight of 1 × 1, $W_{s1}\in R^{3\times 3\times s_{1\times 1}\times e_{3\times 3}}$ is the convolution kernel weight of 3 × 3.

Step 3: The feature maps of the two scales are spliced along the channel dimension, and the channel compression degree is controlled by introducing the compression ratio parameter. Among them, the feature map splicing formula along the channel dimension is:

(7) $$F_{out}=Concat\left(F_{expand1},F_{expand3}\right)$$

**Figure 2:**
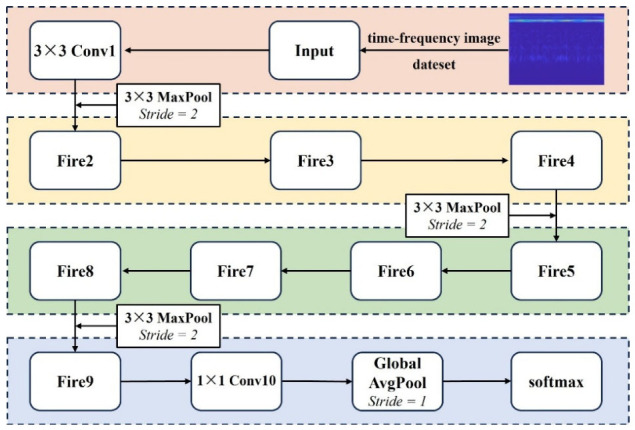
SqueezeNet model architecture.

After introducing the Fire module, SqueezeNet optimized the overall network structure, which significantly reduced the model parameters while still maintaining a strong feature extraction ability. Its overall architecture consists of an input layer, an initial convolution layer, a Fire module stack layer, a global average pooling layer, and a final Softmax classification layer, as shown in Figure 2.

The network architectural design of the SqueezeNet model mainly includes the following parts:

Initial convolution layer: a larger convolution kernel is used to capture more spatial feature information in the input stage, and a convolution operation with a step size of 2 is applied to lower the feature map resolution and boost computational efficiency.

Fire module stacking layer: Multiple Fire modules are stacked in series to form a backbone network, which is responsible for the extraction and expression of deep features. The number and parameter design of Fire modules are optimized to ensure that the network can still maintain strong feature extraction capabilities at low parameter levels.

Maximum pooling: After the Conv1, Fire4 and Fire8 layers, the maximum pooling operation with a step size of 2 is adopted to gradually downsize the feature map, thus decreasing the volume of calculation and improving the calculation efficiency of the model.

Global average pooling layer: The global average pooling layer serves as a substitute for the conventional fully connected layer. The global mean value of the final layer’s feature map is computed, converting the feature map into a fixed-length vector that represents the class probability distribution.

Softmax classification layer: The output of the global average pooling layer finally passes through the Softmax layer to obtain the probability distribution of each category for the final classification decision.

In summary, through the design strategies of the Fire module, large-scale convolution kernel, maximum pooling and global average pooling, the SqueezeNet model can significantly reduce the network parameters while maintaining the same feature expression ability as the deep network, so that it still has excellent classification ability in low computing resource environments.

### 2.3. Transfer Learning Strategy

Transfer learning is a machine learning paradigm that leverages knowledge acquired from the source domain to boost both the efficiency and performance of learning tasks in the target domain. Unlike traditional machine learning, which requires that the training and test datasets follow an independent and identical distribution, transfer learning enables the effective transfer of tool wear knowledge across different working conditions. This is achieved through the sharing of features, model parameters, or task objectives, via mapping operations between the source and target domains [26]. The specific concept is defined as: given a source domain $D_{s}=\left\{X_{s},P\left(X_{s}\right)\right\}$ and a target domain $D_{t}=\left\{X_{t},P\left(X_{t}\right)\right\}$, the learning tasks are $T_{s}=\left\{Y_{s},f_{s}\left(\cdot \right)\right\}$ and $T_{t}=\left\{Y_{t},f_{t}\left(\cdot \right)\right\}$, respectively, and the target prediction function $f_{t}\left(\cdot \right)$ in $D_{t}$ is improved by using the knowledge in $D_{s}$ and $T_{s}$, where $D_{s}\neq D_{t}$ or $T_{s}\neq T_{t}$.

In tool wear status monitoring based on traditional machine learning and deep learning, accurate identification of tool wear conditions depends on large volumes of tool wear sample data and the calibration of network parameters, which involves multi-scale training on abundant tool wear image samples. This ensures that parameters at all levels converge during training, enabling classification results with high accuracy and robustness.

In the condition monitoring of tool wear under variable working conditions, due to the change in working conditions such as the selection of tool-workpiece, the setting of process parameters and the application of cutting fluid, there are obvious differences between the distribution characteristics of tool wear signal data and the underlying wear mechanisms across different working conditions [27]. At this time, if the model trained by the old working condition data is used to identify the tool wear status under the new working condition, the generalization ability of the model will be greatly limited, resulting in poor recognition results of the tool wear status under different working conditions [4]. To this end, this work proposes a phased transfer learning strategy based on the lightweight SqueezeNet model. After using the milling cutter wear data of the old working conditions to train the model completely, fully learning the basic characteristics of the milling cutter wear status, the trained model parameters are used as the initial weights of the transfer learning model. On this basis, for the tool wear signal data from new working conditions, only some layers of the network are fine-tuned to accommodate shifts in feature distribution across different working conditions. This staged transfer learning strategy aims to enhance the classification adaptability of the model under variable working conditions, so that it can maintain good feature transferability on vibration signal data under different working conditions. Compared with retraining or simply mixing multi-condition data, this method can make efficient use of existing knowledge, reduce dependence on a single condition, and improve the robustness and generalization ability of the model. In addition, the phased training enables the model to optimize the model locally for the new working conditions while retaining the basic features learned from the old working conditions, so as to better adapt to the vibration signal distributions present across different working conditions.

### 2.4. Wear Status Monitoring Model of Milling Cutter Under Variable Working Conditions Based on Transfer Learning and Lightweight SqueezeNet

**Figure 3:**
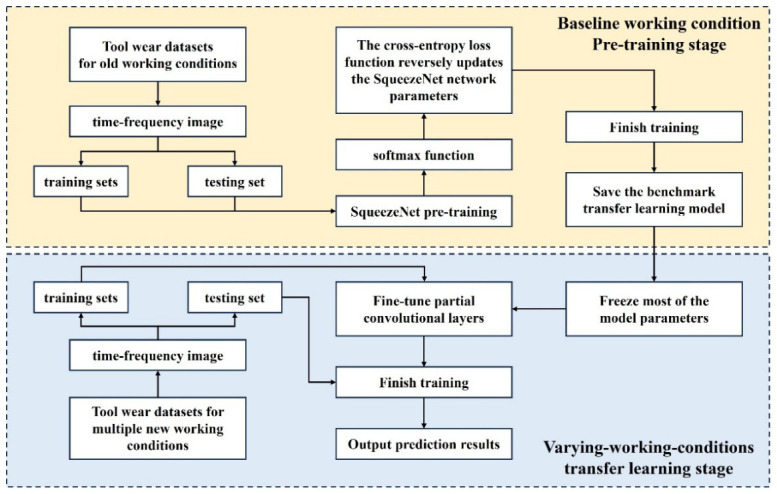
Flow chart of wear status monitoring model of variable working condition milling cutter based on transfer learning and lightweight SqueezeNet.

Aiming at the limitations of conventional machine learning and deep learning approaches in accurately identifying the wear status of small-sample variable condition milling cutters, a wear status monitoring model for variable condition milling cutters based on transfer learning and lightweight SqueezeNet is proposed. Firstly, CWT is used to convert the original vibration signal of milling cutter wear into a time–frequency diagram, so as to completely retain the global time–frequency characteristics and distribution characteristics contained in the signal. Secondly, the pre-trained lightweight SqueezeNet neural network is leveraged to extract robust wear-related features of milling cutters under varying working conditions. Finally, the parameters of the pre-trained model are fine-tuned and optimized by the transfer learning strategy, which further improves the prediction accuracy and robustness of the tool wear status monitoring model across different working conditions, avoids the time and calculation cost of repeated training of the model, and realizes the accurate identification of the wear status of the milling cutter under small sample variable working conditions. The flow chart of the variable condition milling cutter wear status monitoring model based on transfer learning and lightweight SqueezeNet is shown in Figure 3. The specific model construction workflow is detailed as follows.

Firstly, the tool wear data is preprocessed as follows:

- Collect vibration signals associated with tool wear under variable working conditions.
- The one-dimensional time-series vibration signal data is converted into a two-dimensional time–frequency representation via CWT.

Secondly, a lightweight SqueezeNet model is established:

- With the Fire module as the core component, the network input and output dimensions are defined.
- Following the lightweight strategy of 1 × 1 convolution priority, channel compression and pooling layer delay, the core architecture of the SqueezeNet model is constructed.
- The global average pooling is used to replace the fully connected layer to reduce the overall network parameter count.

Finally, combined with the transfer learning strategy, the wear status monitoring model of the milling cutter under variable working conditions is constructed:

- Pre-training: The data of the old working conditions are used for complete training, so that the network can initially learn the characteristics related to the wear status of the milling cutter. After the training is completed, the model parameters after the training of the old working condition data are saved as the initial weight of the transfer learning.

Variable working condition transfer learning: partial data under multiple sets of new working conditions are introduced in turn to fine-tune the model, so that the model can adapt to the feature distribution across diverse working conditions. In this stage, only part of the high-level convolution layer and classification layer of the SqueezeNet are thawed, and the low-level feature extraction part is frozen to retain the basic features learned in the old working conditions. At the same time, the network is allowed to make small-scale adjustments to the data of the new working conditions to adapt to the time–frequency characteristics of tool wear vibration signals under different working conditions.

### 2.5. Model Evaluation Indicators

The evaluation index is an important means to measure the performance of the prediction model, and it is also a key parameter for objectively comparing the proposed model with the benchmark model. The confusion matrix is an important tool for the classification model evaluation, which shows the prediction results of the model on all classes. For multi-classification problems, each element within the confusion matrix corresponds to the intersection of the true label and the predicted label. The core indicators of the classification model prediction results used in this work are the confusion matrix and accuracy. The following is the specific calculation method of accuracy and its significance:

(8) $$Accuracy=\frac{TP+TN}{TP+TN+FP+FN}$$

In the formula, TP denotes positive samples correctly identified by the model, FP refers to negative samples misclassified as positive by the model, FN indicates positive samples misclassified as negative by the model, and TN represents negative samples correctly classified by the model.

## 3. Experimental Verification and Result Analysis

### 3.1. Experimental Design

To validate the performance and reliability of the proposed wear status monitoring model for milling cutters under variable working conditions, a full lifecycle wear test of milling cutters under diverse working conditions was conducted.

The experiment was performed on the TJ-V855 three-axis CNC machine tool (Hunan Taijin CNC Machinery Co., Ltd., Shaoyang, China). The cutting tool selected was a four-flute cemented carbide end mill with dimensions of 10 mm × 75 mm × 30 mm. The workpiece was a 45 # steel cuboid thick plate with a size of 100 mm × 100 mm × 50 mm. The cutting stroke of each tool was 100 mm. The milling process was cooled by coolant. A new milling cutter was used in each working condition until it was completely scrapped. The milling process parameters for the different working conditions are listed in Table 1.

**Figure 4:**
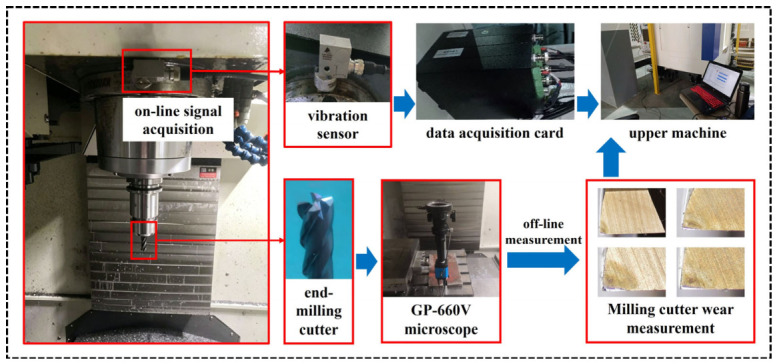
The platform of milling cutter wear experimental.

In the milling process, the PC-ZD-03D triaxial vibration sensor (Shenzhen Gilandin Intelligent Technology Co., Ltd., Shenzhen, China) mounted on the machine tool spindle is employed to acquire the vibration signals along the X, Y, and Z axes of the machine spindle. The sampling frequency is 20 KHz, with data transmitted to the acquisition system through the PK-ZD-VM01 acquisition card (Shenzhen Gilandin Intelligent Technology Co., Ltd., Shenzhen, China). The GP-660V optical microscope (Kunshan Gaopin Precision Instrument Co., Ltd., Kunshan, China) is used to observe the wear of the milling cutter. The magnification of the microscope is 41–270 times. Combined with the supporting measurement software, the accurate measurement of the wear of the milling cutter can be realized, and the resolution can reach 0.001 mm. The whole life wear experimental platform of the milling cutter is shown in Figure 4.

**Figure 5:**
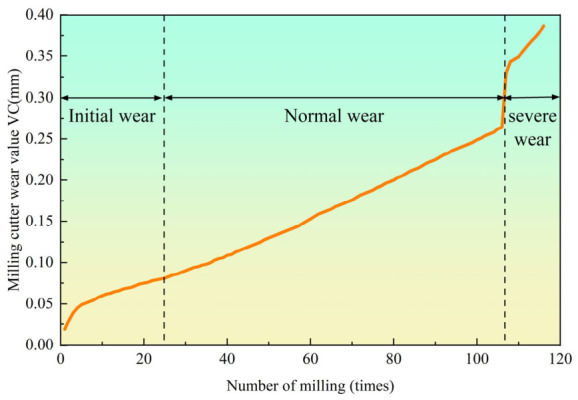
Working condition 1: Wear curve of milling cutter.

However, due to the relatively weak vibration energy of the *Y*-axis vibration signal during the milling process, the ability to characterize the wear status of the milling cutter is weak, so only the milling vibration data from the X and Z axes are used as the model input. The judgment standard of the wear status of the milling cutter is set as the length VC value of the wear area of the milling cutter along the intersection line of the main flank face and the secondary flank face. According to the Chinese national standard GB/T 16460-2016, considering the actual production demand of the enterprise, the milling cutter wear state is divided into three categories: initial wear, normal wear and severe wear [28]. The wear curve of the milling cutter under working condition 1 is shown in Figure 5. When the tool wear VC is in within 0–0.08 mm, the milling cutter was classified as being in the initial wear stage, and the signal data is marked as state “1”; when the tool wear VC is within 0.08–0.3 mm, the milling cutter is judged to be in a normal wear status, marked as state “2”; when the tool wear VC is greater than 0.3 mm, the machining quality decreases obviously. It is judged that the milling cutter is in a serious wear status and marked as state “3”. At this time, prompt tool replacement is required to avoid workpiece rejection due to excessive wear.

Therefore, it is necessary to mark the whole life wear test samples of the three groups of milling cutters under diverse working conditions according to the judgment standard of milling cutter wear status. However, due to the fact that the *X*-axis and *Z*-axis vibration signals are decoupled into two independent input channels in the transfer learning framework, each original sample will generate two independent input samples of *X*-axis and *Z*-axis, respectively. The number of samples in each stage across the three diverse working conditions was doubled relative to the original dataset, as shown in Table 2.

### 3.2. Verification of Wear Status Monitoring Model of Milling Cutter Under Variable Working Conditions Based on Transfer Learning and Lightweight SqueezeNet

#### 3.2.1. CWT Time–Frequency Diagram Extraction

The verification of the model is based on the collected *X*-axis and *Z*-axis vibration data. Firstly, three groups of milling cutter wear vibration datasets collected under diverse working conditions are subjected to the necessary interception and filtering processing, and only the stable milling stage signal within the frequency band of 0–7000 Hz is retained as the effective data. Secondly, the standard deviation S of the vibration signal data is calculated to remove the abnormal points with too large deviation values. Finally, CWT is employed to transform the preprocessed one-dimensional time-series vibration signal into two-dimensional time–frequency representations, thereby generating the time–frequency diagram dataset for the full lifecycle wear of milling cutters across diverse working conditions. The parameter settings of CWT are shown in Table 3.

Among them, through comparative analysis, the bandwidth parameter $F_{b}=3$ and the center frequency $F_{c}=3$ are selected as the parameter combination (that is, the CMOR3-3 wavelet basis function is selected), which can achieve the best balance between time–frequency localization performance and computational efficiency. The symmetry characteristics of CMOR3-3 make the time–frequency distribution of signal data have better visual interpretability, and the generated time–frequency diagram can provide more abundant information.

**Figure 6:**
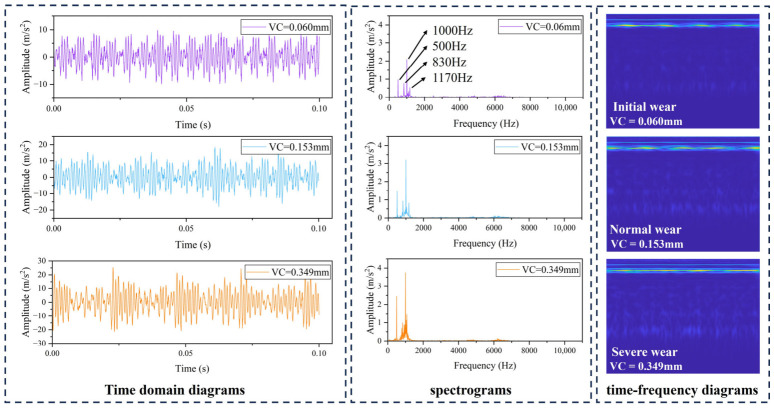
Working condition 1: Time domain diagram, spectrum diagram and time–frequency diagram of vibration signal in different wear stages of the *Z* axis.

The time domain diagram, spectrum diagram and time–frequency diagram of the vibration signal of the *Z*-axis at different wear stages are shown in Figure 6. It can be found from the time domain diagram of the vibration signal that the amplitude of the vibration signal gradually increases with the increase in the wear of the milling cutter and shows a significant positive correlation with the wear amount. From the spectrum diagram, it can be seen that the energy of the low-frequency cutter teeth passing through the frequency increases gradually with the wear of the milling cutter, and concentrates on 500 Hz and 1000 Hz, while the energy of the high-frequency noise increases with the increase in the wear of the milling cutter.

The time–frequency diagram shows the following change trend: with the increase in the wear amount of the milling cutter, the low-frequency energy is enhanced, and the brightness of the low-frequency energy strip is gradually increased and widened; at the same time, more large-area continuous highlight areas appear in the high frequency band, showing a more blurred and diffused form; the overall time–frequency diagram evolves from a regular and clear structure to a more blurred and irregular shape, and the change process of its characterization is highly corresponding to the time domain diagram and the spectrum diagram. Compared with the time domain diagram and the frequency spectrum diagram, the time–frequency diagram can capture the wear characteristics of the time domain and the frequency domain at the same time, which can more intuitively and effectively reflect the state evolution process of the milling cutter wear, and provide more abundant and reliable data support for the recognition of the milling cutter wear state. In addition, the results further verify the effectiveness of the time–frequency diagram as an input to the Squeeze model.

#### 3.2.2. Model Verification

**Figure 7:**
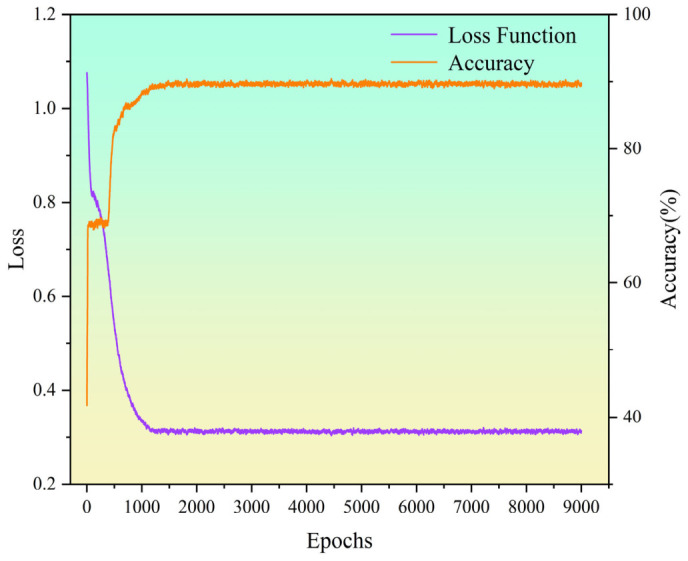
The accuracy and loss function curve of the SqueezeNet model pre-training process.

To validate the prediction performance of the variable condition milling cutter wear monitoring model proposed in this work, the data sequence of the time–frequency diagram dataset of the full lifecycle of milling cutter wear under working condition 1 is randomly shuffled. The SqueezeNet model is then pre-trained by partitioning the input into training and test sets at a 4:1 ratio. The parameter setting of the network is clarified through multiple debugging. It is required that the accuracy of the model continues to increase during the training process, the loss function gradually decreases, and there is no over-fitting phenomenon, so as to ensure that the prediction performance of the model after migration is the best. The network parameters settings of the SqueezeNet model pre-training are listed in Table 4, and the trend of the accuracy and loss function of the training process is shown in Figure 7.

**Figure 8:**
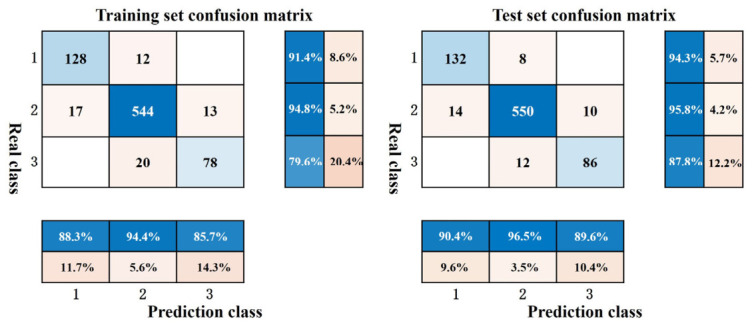
The confusion matrix of the variable working condition milling cutter wear status monitoring model based on transfer learning and SqueezeNet under condition 2.

**Figure 9:**
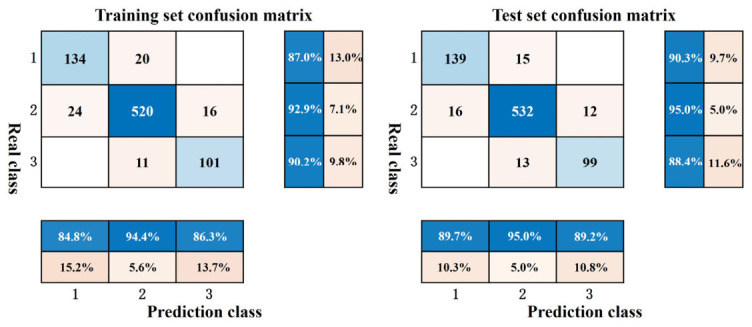
The confusion matrix of the variable working condition milling cutter wear status monitoring model based on transfer learning and SqueezeNet under condition 3.

Then, on the basis of the SqueezeNet pre-training model, the low-level feature extraction part of the network is frozen and only part of the high-level convolution layer and classification layer of SqueezeNet are thawed to retain the basic features learned by the model in working condition 1, so as to construct the wear status monitoring model of the variable working condition milling cutter. The time–frequency diagram datasets of condition 2 and condition 3 are introduced in turn, and they are partitioned into training and test sets at a 1:1 ratio, and then input into the model. This division method is different from the 4:1 division of the pre-trained dataset, primarily to assess the model’s generalization ability on unseen data more evenly and mitigate the impact of training data bias. The prediction results of the confusion matrix of the training set and the test set of the variable working condition milling cutter wear status monitoring model based on transfer learning and SqueezeNet under working conditions 2 and 3 are shown in Figure 8 and Figure 9.

Under working condition 2, 12 groups of samples were misjudged as normal wear in the initial wear of the model training set, and the misjudgment rate was 8.6%. There were 17 and 13 samples of normal wear that were misjudged as initial wear and severe wear, yielding misjudgment rates of 3% and 2.2%, respectively. In severe wear, 20 groups of samples were misjudged as normal wear, with an associated misjudgment rate of 20.4%. In the initial wear test set, 8 groups of samples were misjudged as normal wear, with a misjudgment rate of 5.7%. For normal wear, 14 and 10 samples were misjudged as initial wear and severe wear, with misjudgment rates of 2.4% and 1.8%, respectively. Twelve groups of samples with severe wear were misjudged as normal wear, and the misjudgment rate was 12.2%.

Under working condition 3, 20 groups of samples were misjudged as normal wear in the initial wear of the model training set, and the misjudgment rate was 13.0%. There were 24 and 16 groups of samples that were misjudged as initial wear and severe wear, and the misjudgment rates were 4.3% and 2.8%, respectively. Serious wear: 11 groups of samples were misjudged as normal wear; the misjudgment rate was 9.8%. In the initial wear test set, 15 groups of samples were misjudged as normal wear, with a misjudgment rate of 9.7%. For normal wear, 16 and 12 samples were misjudged as initial wear and severe wear, with misjudgment rates of 2.9% and 2.1%, respectively. A total of 13 severe wear samples were misjudged as normal wear, and the misjudgment rate was 11.6%.

On the whole, this work presents a variable condition milling cutter wear status monitoring model based on transfer learning and the SqueezeNet. The accuracy of milling cutter wear status recognition across diverse milling conditions is relatively close and both are at a high level. Among them, the recognition accuracy of the training and the test set under condition 2 is 92.365% and 94.581%, respectively; the recognition accuracy of the training and the test set under condition 3 is 91.404% and 93.220%, respectively.

#### 3.2.3. Comparative Study

**Figure 10:**
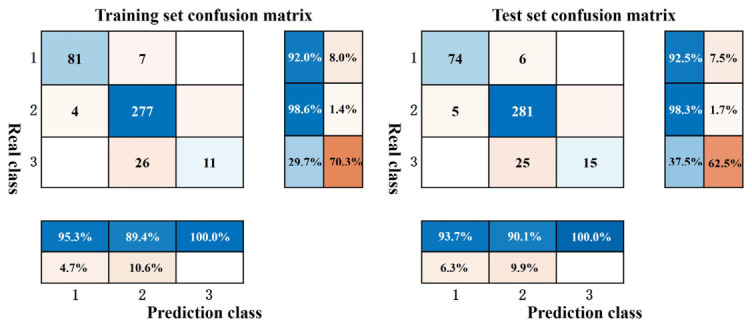
The confusion matrix of LSTM-DBO-SVM milling cutter wear status monitoring model under condition 2.

**Figure 11:**
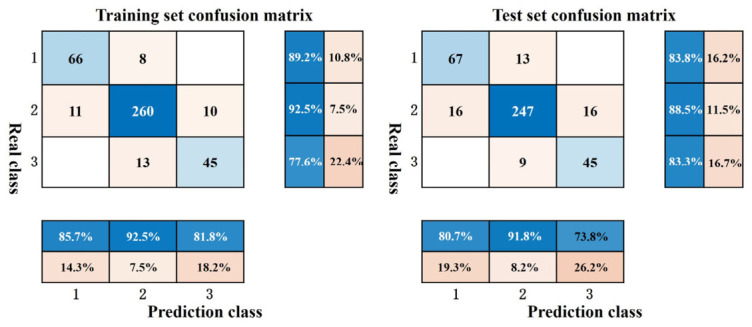
The confusion matrix of LSTM-DBO-SVM milling cutter wear status monitoring model under condition 3.

To further validate the advantages of the variable working condition milling cutter wear status monitoring model based on transfer learning and SqueezeNet proposed in this work, the LSTM-DBO-SVM milling cutter wear status monitoring model proposed in the literature [29] is trained and tested on the same dataset, and compared for comparative evaluation against the proposed method. Among them, the LSTM-DBO-SVM milling cutter wear status monitoring model parameter settings are shown in Table 5, and the classification results of the training and test set confusion matrix under condition 2 and condition 3 are shown in Figure 10 and Figure 11.

As illustrated in Figure 9, under condition 2, the prediction accuracy achieved by the LSTM-DBO-SVM model test set for initial wear is 92.5%, while it is poor in the classification performance of severe wear, and about 62.5% of the samples are misjudged as normal wear. This indicates that the model is not sensitive to the severe wear stage under the second working condition. The possible reasons are as follows: the time-series model has a lag effect on the capture of nonlinear degradation characteristics in the vibration signal; with the change in working condition parameters, the overlapping degree of time domain features in different wear stages increases, thus reducing the accuracy of prediction. It is worth noting that the sample count of state “3” in condition 2 is relatively small, so although its misjudgment rate is high, its impact on the overall classification accuracy is still limited.

By comparing Figure 7 and Figure 9, it can be seen that the variable working condition milling cutter wear status monitoring model based on transfer learning and SqueezeNet shows a more balanced classification performance under working condition 2. The recognition accuracy of the state “3” on the test set can reach 87.8%, which is about 50.3% higher than that of the LSTM-DBO-SVM model. This is mainly due to the spectral resolution of the time–frequency diagram, which can clearly reflect the wide-band energy diffusion characteristics unique to the severe wear stage of the milling cutter. The deep convolution structure of SqueezeNet has strong representation ability in the extraction of spatial-frequency joint features.

By comparing Figure 8 and Figure 10, the difference in classification performance between the LSTM-DBO-SVM model and the model developed in this work under condition 3 can be highlighted. In the early wear stage of the test set, about 16.2% of the samples were misjudged as state “2”, and the recognition accuracy was about 4.21% lower than that of condition 2. This performance degradation is mainly due to the lack of sensitivity of the model to the change in working conditions. Especially when the process parameters change, the statistical characteristics of the vibration signal will shift, and the stability of the sensitive feature set will decrease. The latter can still maintain a test set accuracy of 93.220% under condition 3, and the misjudgment rate in the severe wear stage is only 11.6%, showing stronger generalization ability. This advantage is mainly due to the good coding ability of the pre-training network for time–frequency features and the cross-condition adaptability provided by the parameter adaptive strategy in the fine-tuning stage, so that the model can still obtain higher recognition accuracy under variable working conditions.

In order to compare the advantages and disadvantages of the prediction model more accurately, this work uses the accuracy index to evaluate the performance of the model. A higher prediction accuracy indicates superior classification performance. Table 6 shows the comparison of the prediction accuracy of the two models under condition 2 and condition 3 and further quantifies their classification ability in the variable condition.

In general, the classification accuracy of the model proposed in this work outperforms that of the LSTM-DBO-SVM model under conditions 2 and 3, especially on the test set. Among them, in condition 2, the accuracy of the test set of the model proposed in this work is 3.448% higher than that of LSTM-DBO-SVM; in the third condition, the accuracy of the test set of the proposed model is increased by 6.295%. This result shows that the wear status monitoring model of the milling cutter under variable working conditions with a time–frequency map as input can maintain high classification performance under variable working conditions and has better generalization ability.

From the perspective of recognition accuracy, the training effects of the two methods in condition 2 and condition 3 are close but the accuracy of the model proposed in this work is still slightly higher than that of the LSTM-DBO-SVM model. This shows that although the model only uses the pre-training weight of condition 1 and adapts to the new condition data by fine-tuning, its classification ability is still strong, which can fully extract key features and enhance the generalization performance of the model. When the LSTM-DBO-SVM model is directly applied to the new working condition data, its generalization ability is limited to a certain extent, resulting in a decrease in the classification effect on the test set, especially in the third working condition.

From the comparison of the confusion matrix, the classification performance of the model proposed in this work is more stable under different milling cutter wear conditions, and the misclassification situation is relatively less, indicating that it exhibits strong adaptability to new working condition data. The LSTM-DBO-SVM model has a higher misclassification rate in the severe wear category, and the proposed recognition effect in this category is better. This may be due to the more intuitive feature expression of the time–frequency map in the severe wear stage, which enables the model to extract key features more accurately.

In summary, the wear status monitoring model of the variable condition milling cutter based on transfer learning and SqueezeNet proposed in this work has stronger classification ability in a variable condition environment. It can not only achieve higher accuracy on the training set but also has better generalization ability on the test set than the LSTM-DBO-SVM model. Therefore, this method is more suitable for the milling cutter wear status monitoring task in this work, especially in the variable working condition scenario; its performance advantage is more obvious, which provides an important reference for the subsequent intelligent monitoring research of milling cutter wear condition.

## 4. Conclusions

Aiming at the problem that the difference in signal feature distribution under different machining conditions leads to insufficient generalization ability of the tool wear status monitoring model and low accuracy of wear status recognition, a method of tool wear status monitoring under variable working conditions based on transfer learning and SqueezeNet neural network is proposed. This method mainly uses CWT to conduct time–frequency conversion processing on the milling cutter wear vibration signal to completely retain the joint feature distribution characteristics of the signal in the time–frequency dimensions. The lightweight SqueezeNet convolutional neural network is used to extract key features, and combined with the phased model transfer learning strategy, enables adaptive and accurate identification of milling cutter wear states across variable working conditions. The proposed method is validated through experiments, with key findings summarized as follows:

(1) The recognition accuracy of the milling cutter wear status monitoring model proposed by this method is close to each other under different working conditions, and they are all at a high level. Specifically, the training and test set accuracies under working condition 2 reach 92.365% and 94.581%; under working condition 3, the corresponding accuracies are 91.404% and 93.220%.
(2) Compared with the LSTM-DBO-SVM model, the milling cutter wear status monitoring model proposed by this method has higher recognition accuracy of the test set under variable working conditions. The recognition accuracy of the test set under condition 2 and condition 3 is increased by 3.448% and 6.295%, respectively, showing stronger generalization ability and stability.
(3) The recognition accuracy of the milling cutter wear status monitoring model proposed by this method in the severe wear stage of the milling cutter is significantly higher than that of the LSTM-DBO-SVM model, which shows that the time–frequency diagram obtained by CWT can more intuitively express the wear characteristics of the milling cutter, thereby enhancing the adaptability of the model to different working conditions.

## Figures and Tables

**Table 1:** Milling cutter wear experiment processing parameters.

Working Condition	Spindle Speed/(r/min)	Feed per Tooth/(mm/z)	Cutting Depth/(mm)	Cutting Width/(mm)
Condition 1	2500	0.25	0.2	7
Condition 2	2750	0.25	0.2	7
Condition 3	3000	0.25	0.2	7

**Table 2:** The number distribution of samples in each wear stage under different working conditions.

Working Condition	Number/Proportion of Initial Wear Samples	Number/Proportion of Initial Wear Samples	Number/Proportion of Normal Wear Samples
Condition 1	336/20.7%	1134/69.8%	154/9.5%
Condition 2	280/17.2%	1148/70.7%	196/12.1%
Condition 3	308/18.5%	1120/67.1%	240/14.4%

**Table 3:** CWT parameter settings.

Parameter	Set Value	Parameter	Set Value	Parameter	Set Value
bandwidth parameter	3	wavelet scale sequence	1–256	picture resolution	300 dpi
center frequency	3	time translation	50 μm	amplitude normalization range	[0, 1]

**Table 4:** SqueezeNet model pre-training network parameter settings.

Parameter	Set Value	Parameter	Set Value	Parameter	Set Value
Optimizer	Adam	Maximum number of training epochs	20	Number of classifier outputs	3
Initial learning rate	0.001	Batch size	64	Loss function	Cross entropy

**Table 5:** Parameter setting of LSTM-DBO-SVM milling cutter wear status monitoring model.

Algorithm	Parameter	Set Value	Parameter		Set Value	
DBO-SVM	Population size	22	Upper bound of search space boundary		[10, 2^8^]	
	Iteration times	30	Lower bound of search space boundary		[0.1, 0.3125]	
SVM	Kernel function	Radial basis function (RBF)	Regularization penalty coefficient	0.5523	Bandwidth parameters of Gaussian kernel function	0.313
LSTM	Hidden unit	30	Activation function	Sigmoid	Unit state activation function	tanh
Optimizer	L2 regularization coefficient	0.01	Initial learning rate	0.001	Optimistic algorithm	Adam

**Table 6:** Comparison of classification accuracy of the two models under different working conditions.

Evaluation Index	LSTM-DBO-SVM Milling Cutter Wear Status Monitoring Model (Condition 2)	The Milling Cutter Wear Status Monitoring Model Proposed in This Work (Condition 2)	LSTM-DBO-SVM Milling Cutter Wear Status Monitoring Model (Condition 3)	The Milling Cutter Wear Status Monitoring Model Proposed in This Work (Condition 3)
Training set accuracy (%)	90.887	92.365	89.831	91.404
Test set accuracy (%)	91.133	94.581	86.925	93.220

## Data Availability

The original contributions presented in this study are included in the article. Further inquiries can be directed to the corresponding author.
